# Perceived Impacts of the Expanded Child Tax Credit Cessation Based on Family Income Level

**DOI:** 10.21203/rs.3.rs-4572262/v1

**Published:** 2024-06-28

**Authors:** Elizabeth Adams, Annika Krovi, Abigail Berquist, Peyton Mosher, Roddrick Dugger, Tegwyn Brickhouse, Melanie Bean

**Affiliations:** University of South Carolina; J.L. Mann Academy of Mathematics, Science, and Technology; University of South Carolina; University of South Carolina; University of South Carolina; Virginia Commonwealth University; Virginia Commonwealth University

**Keywords:** Poverty, tax credit, low income, child health, COVID-19

## Abstract

The 2021 Expanded Child Tax Credit (ECTC) lifted millions of children out of poverty and drastically improved well-being. These impacts were particularly salient for families with lower income among those who received the full ECTC benefit. This study gathered lived experiences on the ECTC cessation and explored differential impacts across income levels to inform discussions around policy restoration. Semi-structured interviews were conducted with parents who had a child (2–12 years) who received the full ECTC. Interviews occurred in May 2022 after the ECTC ended. Changes in financial security and health were assessed. Families were classified as having lower vs. higher income (LI; n=19 vs. HI; n=19) corresponding to household income below vs. above 200% of the Federal Poverty Line. Inductive analysis and constant-comparison methods generated themes on similarities/differences between groups. Results indicated families with LI experienced severe financial constraints and greater negative emotions, after the ECTC ended. Many reduced spending, budgeted, accepted financial support from family/friends, and delayed credit card payments. More families with HI reported the ECTC provided a financial buffer placingthem in a more secure position to meet current needs. Both groups reported negative impacts from inflation coinciding with the ECTC ending and minimal changes in their income tax return. Families overwhelmingly reported a desire for the ECTC to continue, despite experiencing different degrees of impact due to these financial changes. Families with LI faced greater hardships after the ECTC ended. Differences across income highlight the need for ECTC restoration, particularly for families in severely under-resourced circumstances.

## INTRODUCTION

The COVID-19 pandemic exacerbated financial hardships and widened health inequities for families with low financial security [1]. In light of this, pandemic relief packages introduced novel policy changes to provide economic support, including the expansion of the Child Tax Credit (CTC). Prior to COVID-19, the CTC included an annual lump sum up to $2,000/child for eligible families as a tax break to offset the costs of raising a child [2]. Tax credits, such as the CTC, greatly reduce poverty and improve educational outcomes [3, 4]; yet, substantial shortcomings of the CTC limited its impact on reducing poverty and advancing health equity. The annual payment structure did not facilitate the use of CTC funds on more immediate basic needs (e.g., food; rent) [2], and excluded households with an income below the taxable amount; thus, some Black, Hispanic, single-parent households, and those living in rural areas were less likely to benefit [5].

In the American Rescue Plan Act of 2021 [6], the expanded Child Tax Credit (ECTC) drastically improved the CTC policy for participating families [6]. The eligibility threshold was widened and the income floor removed, thus allowing more families to qualify. For the first time, the ECTC included the lowest earning families, as well families earning up to $112,500/year (single parent homes) and $150,000/year (dual earning homes). The total value of the credit increased to $3,000/child (ages 6–17 years) and $3,600/child (ages 6 years and younger), resulting in historic levels of financial relief. The payment structure was modified, such that half the funds were disbursed as unconditional monthly payments from July to December 2021, and the other half remained an annual tax break. These changes led to profound reductions in child poverty, improved health outcomes, greater income stability, and educational investments in minoritized communities [7–9]. Yet, these benefits were temporary, as the cessation of the ECTC resulted in greater financial strain, unmet basic needs, and rising child poverty rates [9–11].

Previous work from our team included interviews with families when receiving the ECTC monthly payments in Fall 2021 [12]. This sample consisted of families across the income spectra who all received the full ECTC benefit. Families reported the ECTC monthly payments improved their financial security, emotional capacity, and overall well-being; yet the lowest earning families experienced these benefits to a greater extent. The ECTC monthly payments facilitated families’ abilities to meet their basic needs, whereas those with relatively higher incomes could afford their basic needs and thus invested ECTC monthly payments in more quality-of-life improvements. Now that the ECTC has ended, it is likely that families lower on the income spectra were disproportionately impacted by the ECTC cessation.

The purpose of this study was to conduct a follow-up interview with the same families to understand their lived experiences after the ECTC ended. This follow-up interview aimed to elicit perspectives on parent’s financial security and physical and emotional well-being after the ECTC ended. Similarities and differences in the experiences of families who were lower versus higher on the income spectra among families earning the full benefit were derived to inform ongoing political discussions around the restoration of the ECTC.

## METHODS

### Study Design

This study used a phenomenological qualitative design. Interviews were conducted in Spring 2022, after the ECTC monthly payments ended and families received their income tax return. Parents shared their perceptions on the ECTC policy and its cessation. Topics included financial security, food access, medical care, and emotional well-being. All procedures were approved by the university’s institutional review board. The Consolidated Criteria for Reporting Qualitative Research (COREQ) was used [13].

### Sampling and Recruitment

Participants were recruited from a larger observational study on the ECTC [14]. Recruitment methods used electronic medical records at an academic medical center in the southeastern United States. Data queries scanned medical records to identify households with a parent ≥ 18 years and a child 2–12 years of age. Contact information was extracted, and parents were emailed to identify their interest in participating. Interested parents completed a REDCap [15] screener to ensure they qualified for the full ETC benefit. See Adams et al [14].

After completing a baseline survey, enrolled parents were emailed about the opportunity to participate in two sequential phone interviews. Of those interested (n = 192); n = 40 were contacted to provide verbal consent. Parents were purposefully sampled based on annual household income, as reported in the baseline survey. Two groups were created: families (n = 19) earning < 200% of the Federal Poverty Line (FPL) [16] and families (n = 21) earning > 200% of the FPL. Groups were labeled as lower income (LI) vs. higher income (HI), respectively, yet all qualified for the full ECTC benefit. A racially proportionate sample within each income group was purposively selected to reflect demographics of the recruitment location (50% black; 50% white). Two families were lost to follow-up for the second interview; thus, data presented here represent 38 families (n = 19 LI; n = 19 HI) who completed a second interview.

### Qualitative Measures

Semi-structured phone interviews were conducted in May 2022, corresponding to five months after ECTC monthly payments ended and after families filed income taxes. The interview guide was adapted from our previous work [12]. Questions asked about perceived financial impacts of the ECTC ending, coping strategies, access to food, mental health, income tax returns, and general opinions on the ECTC (See Appendix for interview guide).

### Qualitative Interviews

#### Preliminary testing

The interview guide was refined through peer debriefing and pilot interviews with families not enrolled in this study [17]. Feedback from pilot interviews (n = 2) improved the clarity of interview questions. For example, the introduction was streamlined and questions about other assistance benefits were revised to capture an adequate understanding of a family’s history of receiving other benefits.

#### Primary interviews

Prior to data collection, positionality statements were written by investigators to identify, acknowledge, and separate personal experiences from data collection and analysis [18]. Two researchers (AB; ELA) trained in qualitative methodology conducted one-on-one interviews with parents (~ 20-minute duration) between May and July 2022. Interviewers were female public health researchers with no previous relationships with study participants.

#### Data Analysis

Interviews were recorded and transcribed using Otter.ai. Corrected transcripts were uploaded into NVIVO 12. Inductive methods using an immersion crystallization approach were used for analysis. Two trained researchers (AB; PM) independently coded all transcripts and created a codebook. Regular meetings were held between the two coders and a third researcher (ELA) to resolve discrepancies and reach a consensus. Data saturation was established using a “code meaning” approach (interview #12 for the LI group; interview #11 for the HI group) [19, 20]. After coding, researchers re-reviewed all transcripts to ensure no codes were missed. Constant comparison methodology[21, 22] was used to generate themes via collaborative discussions on similarities/differences across income groups [23]. Themes were reviewed and verified using peer debriefing (by AK) to ensure data consistency and integrity.

## RESULTS

Parent and child demographics are in Table 1. Qualitative findings are presented as the impacts of the ECTC cessation on family’s financial security, income tax return, emotional and material resources, and overall views of the ECTC. Within each domain, themes represent similarities and differences between income groups. Representative quotes are presented in Tables 2 and 3.

### Domain 1: Financial security

#### Theme: Financial strain vs. financial buffer

After the ECTC ended, LI families experienced significant financial constraints. Some were not able to meet basic needs (e.g., housing, utilities), while most could not afford occasional needs (e.g., medical bills, car repairs) or quality-of-life spending (e.g., family leisure activities). HI families were more likely to mention the monthly payments provided a financial buffer and placed them in a more secure position to meet their routine and occasional needs. Across the HI group, there was a mix of families who reduced quality of life-spending and those who did not experience substantial financial impacts. In both groups, families attributed inflation as exacerbating their current financial cons9aints whereby rising prices, particularly in food and gas, made routine expenses more challenging to afford. Notably, LI families were more likely to mention inflation as a financial challenge that further exacerbated meeting basic needs.

#### Theme: Degree of financial coping strategies

Groups differed on the type of coping strategies used to adapt to the ECTC ending. LI families prioritized expenses and focused on necessities, such as housing and food. After covering basic needs, many parents determined what spending on items besides necessities, if any, would be most beneficial. In general, budgeting and prioritization were heavily used to combat financial strain. Many LI families also mentioned having to delay credit card payments, out of necessity, and some increased use of other resources (e.g., federal nutrition assistance programs) or accepted financial support from family and friends. Fewer HI families mentioned needing to delay credit card payments, while a few mentioned getting a second job to improve their financial situation. Both groups mentioned prioritizing expenses and reducing quality-of-life spending; yet, LI families did so to a greater extent. A few parents in both groups mentioned spending more time at home to limit outside activities that were financially draining. Overall, most families found these strategies to be helpful and effective in adjusting to the reduced income, yet some LI families still struggled to meet their basic needs, despite budgeting and reducing their routine spending.

### Domain 2: Income tax return

#### Theme: Mixed impacts, yet positive feelings overall

There were mostly similarities across groups related to impacts on their income tax return. Compared to the year prior, families in both groups noticed a smaller tax return or no changes, while a few HI families mentioned receiving a larger tax return amount. For many, it was challenging to disentangle the financial impact on their income tax return that was attributable to the ECTC versus other factors (e.g., changes in employment). Overall, most families had positive or neutral feelings about the ECTC and their income tax return and cited this impact as meeting or exceeding expectations.

### Domain 3: Emotional and material resources

#### Theme: Extent of emotional strain and constrained choices

LI parents experienced greater impacts on their emotional well-being and family relationships due to the cessation of monthly payments. Most reported limited funds and constrained choices resulted in increased negative emotions, such as stress and anxiety. Only a few HI families experienced these negative emotions and most reported no impact on their family relationships. Families in both groups mentioned more budgeting discussions within their families to cope with reduced funds.

#### Theme: Comprehensive vs. minimal changes in food spending

Families in both groups reported limiting food spending after the ECTC ended, yet this was much more prevalent in the LI group. These families reduced the quantity and/or quality of food purchases, ate out less, or budgeted more due to financial constraints. Few HI families reduced the amount and quality of their food purchases, and a majority reported no changes in food spending. Both groups mentioned inflation as impacting their food spending, yet this was a greater barrier in LI families.

#### Theme: Presence vs. absence of barriers to medical care

Many LI families experienced cost barriers to medical care, such as limited transportation or challenges paying for medical expenses. Most HI families did not report cost-related barriers. In both groups, some families mentioned non-cost barriers, such as limited availability of providers and appointments, particularly for mental health services.

### Overall views on ECTC

#### Theme: Desire for ECTC to continue

Overwhelmingly, families in both groups reported a desire for the ECTC to continue, despite experiencing different degrees of impact due to these financial changes. Almost all LI families felt the ECTC was helpful, and HI families often mentioned the ECTC was helpful to others with fewer resources. Most LI families preferred the ECTC over the previous CTC policy, while HI families felt grateful for the ECTC while it lasted.

#### Theme: Anticipated financial security vs. flexibility

Families were asked to project the expected impacts, if the ECTC monthly payments had continued beyond 6 months. More LI families predicted greater financial security and an ability to meet routine needs that would have improved emotional health. More HI families predicted greater financial flexibility and affordability for medical care expenses, primarily dental work. Families in both groups felt their financial security would have improved by meeting occasional needs, quality-of-life spending, greater savings, and reduced debt.

## DISCUSSION

This study documented the lived experiences of families following the cessation of the ECTC. Parents experienced negative emotions, constrained financial choices, and stress to varying degrees after the monthly payments ended. LI parents faced greater financial barriers and felt these impacts more acutely. The onset of inflation coincided with the ECTC payments ending and exacerbated these struggles. Overall, families appreciated the ECTC while it lasted and expressed a desire for it to continue.

A key finding in this study was the differential impacts on family’s financial security. When the ECTC monthly payments ended, families in both groups displayed adaptive resilience by reducing quality-of-life spending and prioritizing expenses. Yet, the LI group struggled to pay for their basic needs, and often felt their financial coping strategies were not enough to combat the negative impacts of no longer receiving these monthly payments. More HI families felt the monthly payments provided a financial buffer and rebuilt their savings, thus suggesting the short-term duration of the ECTC may have been sufficient. These findings are important for policymakers, as discussions around the ECTC restoration continue [24]. A common concern is the financial costs of supporting this policy long-term [25]; therefore, these findings can inform potential modifications to the eligibility criteria of the ECTC. As its cessation impacted LI families to a greater extent, the option of lowering the income cap of eligible families can be explored, in alignment with other proposed modifications for the future of the ECTC [26]. At the same time, the pressing need for LI families to access this financial support was clear, thus maintaining the removal of the income floor is critical to ensuring the lowest resourced families have financial support to afford basic needs. Some families also mentioned a lower use of other federal assistance benefits when receiving the ECTC monthly payments, thus the cost of providing the ECTC long-term should consider the potential offset in costs to other federal assistance programs.

Another key finding was the differential impacts on family’s physical and mental health. Most LI parents experienced emotional strain when the ECTC ended and strong impacts on their emotional well-being. Prior research has shown the ECTC had small benefits on parent mental health [27], yet these qualitative findings indicate far more immediate and significant effects than what was previously understood. When asked about factors that influence their physical health, LI families reported more cost barriers to medical care (e.g., transporting, visit fees) and limited food spending that reduced the quantity and quality of their food. These findings complement previous reports on the ECTC and associations with food security and health status [10, 14, 28] while highlighting the importance of the ECTC and its potential to address the root causes of food insecurity and associated health outcomes by offering flexible financial support. Notably, he ECTCs unconditional nature offers the flexibility needed to address varying physical, medical, and mental health needs.

Overwhelming support for the ECTC, among both LI and HI subsets, was notable. Parents in both groups preferred the ECTC monthly payments over the previous annual lump sum. When the ECTC was first introduced, it was unknown how families would feel about receiving a lower income tax return, due to the advanced monthly payments. Tax refunds are often used to build savings, pay down debt, catch up on overdue bills, or pay for large purchases [29–31]; thus, reducing this amount could negatively impact existing financial planning strategies. Yet, both groups almost exclusively reported positive or neutral feelings about their income tax return, with most reporting a slight decline or no change in the amount they received. Collectively, these findings indicate a strong preference for the novel ECTC structure and support the prospect that many families would opt-in to the monthly payments, if afforded the opportunity to do so again in the future.

An important contextual factor that was raised by both groups was the recognition that inflation was occurring, as the monthly payments were ending. These rising prices caused a greater financial burden that were felt more deeply by LI families and resulted in challenges affording basic needs. These findings add to the exiting literature on the potential for the ECTC to improve family’s financial flexibility, and reinstating this monthly payment structure could offer a strategy to buffer against inevitable economic fluctuations that drive inflation and limit the financial capabilities for LI families.

The strengths of this study include the use of rigorous qualitative methods to elevate the voices of impacted families, a study design built upon previous interviews collected during the ECTC monthly payment period, and timely data to inform ongoing policy discussion regarding the restoration of the ECTC. Limitations include a lack of generalizability to some marginalized groups (e.g., Latino, Asian, Indigenous persons), a minimal distinction between LI and HI families who resided near the income threshold (200% FPL), and a history effect that cannot disentangle the unique financial impacts attributable to inflation versus the ECTC ending. Supportive quantitative data on spending patterns and objective health outcomes could better supplement these qualitative findings to ensure accuracy and comprehensiveness. Nevertheless, this study offers detailed insights into the effects of ending the ECTC payments on families across different income levels.

## CONCLUSION

Child tax credits are a vital way for policymakers to support families in a way that allows children thrive, now and in the future. The ECTC demonstrated this possibility with far reaching impacts across the income spectra. Its cessation solidified its essential value by demonstrating negative physical, emotional, and financial changes for LI families experiencing numerous hardships after the monthly payments ended. These data support ongoing efforts to restore the ECTC at the federal level and similar state-level policies to support families in severely under-resourced circumstances. Overall, these findings provide novel data on the personal impacts of the ECTC and in-depth comparisons between different income groups affected to highlight the importance of this policy and its restoration on family health and well-being.

## Figures and Tables

**Table 1:** Demographics of participating parents (n = 38) and their child. Data presented for the full sample and by income classification.

	All parents (n = 38)	Lower income (n = 19)	Higher income (n = 19)
Parent age, years (mean ± SD)	37.1 ± 6.0	37.2 ± 4.9	36.9 ± 7.1
Child age, years (mean ± SD)	6.2 ± 2.9	6.1 ± 3.0	6.3 ± 2.8
Parent sex, female (%)	97.4	94.7	100
Parent race (%)			
Black	50.0	52.6	47.4
White	50.0	47.4	52.6
Parent ethnicity, Hispanic (%)	2.6	0	5.3
Number of children in home			
1	16	5	11
2	10	6	4
3+	12	8	4
Monthly Income (n)			
<$2,000	5	5	0
$2,000–$2,999	9	7	2
$3,000–$3,999	8	2	6
$4,000–4,999	6	5	1
$5,000–5,999	5	0	5
$6,000–6,999+	5	0	5

* FPL = Federal Poverty Line

**Table 2:** Themes presented within each of the 4 domains. Representative quotes for lower income (n = 19) and higher income (n = 19) families represent their similarities

				Similarities	
Theme			Description	Lower income^a^	Higher income^b^
**1. Financial security**					
Financial strain vs. financial buffer		Families felt inflation caused financial strain	*“It has been a little bit tougher because of inflation right now. Gas prices have gone up. Food has gone up. That extra money would come in handy.”*	*“Between gas prices rising and not having a little extra income, I think we are starting to think twice about certain trips.”*	
Degree of financial coping strategies		Families reported reduced their quality-of-life spending	*“I have had to make some cuts with certain things, like not splurging as much on material items.”*	*“Making the choice to spend wisely because there is not that little bit there to do all those extra things that we like to do for ourselves.”*	
**2. Income tax return**					
Mixed impacts, yet positive feelings overall		Families had positive or neutral feelings about their income tax return	*“It was not too much of a difference. It really did not bother us to get a little less back this year.”*	*“I tried to go in with the expectation that it would not be similar to previous years, and then when it was, it was a nice breath of relief.”*	
**3. Emotional and material resources**					
Extent of emotional strain and constrained choices		Families reported more budgeting discussions with family members	*“Budget conversation have definitely had to take place for us to be able to still keep our wallet a well-oiled machine.”*	*“[The children] are like, ‘Why can we not go here?’ I am like, ‘I do not have [the money] right now. You will have to wait.’ I let them know I am not able to. I do not try to get their hopes up”*	
Comprehensive vs. minimal changes in food spending		Inflation negatively impacted food budgets	*“I feel like the price of food has gone up two or three times since last year. It makes it hard to choose the healthier option because you do not have a lot to stretch.”*	*“Between gas, inflation, and groceries, it is kind of like the payments stopped and everything else skyrocketed out of the blue.”*
Presence vs. absence of barriers to medical care		Limited provider availability was a barrier, particularly for mental heath services	*“It is impossible to find treatment for mental health right now. My children have some issues, and I can not even find them counselors or help or anything.”*	*“There is no one to help anyone. I have called every counseling place within a 40-mile radius of us. I cannot find any counseling or treatment for either of my children.”*
**4. Overall views on ECTC**					
Desire for ECTC to continue	Most families wanted the ECTC to continue, for theirs and others’ benefit	*“I am pretty sure me and every American would love to have the monthly payments back. It is a big help whether you need it or not. If you do not need it, you can put it away for savings. But like I said, the average person needs it.”*	*“I think it should continue for anybody who qualifies, because even if it is not an immediate blessing to them, like for us, our kids will be able to maybe go to nicer colleges. There is a little extra there.”*		
Anticipated financial security vs. flexibility	Predicted improved financial security if ECTC had continued	*“I would be a little more stable, and we would be able to do more things as a family to help with mental health.”*	*“I would have had a little extra money to make sure we had the necessities.”*		

a Families earning 200% Federal Poverty Line;

b Families earning > 200% Federal Poverty Line

**Table 3:** Themes presented within each of the 4 domains. Representative quotes for lower income (n = 19) and higher income (n = 19) families represent their differences.

				Differences	
Theme			Description	Lower income^a^	Higher income^b^
**1. Financial security**					
Financial strain vs. financial buffer		LI experienced financial constraints. HI reported having a financial buffer.	*“Between gas and food, it is really tight. Just household products like toilet paper and everything else.”*	*“The payments worked while we had them, and now we are in a place where we do not necessarily need them anymore.”*	
Degree of financial coping strategies		Groups differed on their type of coping strategies	*“It is just running up on a credit card, which is unfortunate. ALL I can really afford is to make is the minimum [payments]. I am having to wait until tax time to pay the balance off.”*	*“We are trying to be safe an trying to save. I may have to pick up a second job for the time just to get afloat and catch up.”*	
**2. Income tax return**					
Mixed impacts, yet positive feelings overall		A few HI reported a larger income tax return	*“I think it [tax return] was just a little bit less. Not a significant amount.”*	*“I got a bigger refund because of the Child Tax Credit and other COVID relief funds, because I made less money since COVID hit. They went off my earned income credit from two years ago. Both of them made a big difference.”*	
**3. Emotional and material resources**					
Extent of emotional strain and constrained choices		LI experienced greater negative impacts on emotional well-being	*“I think I stress a bit more about the financial aspect of it.”*	*“No stress. I would like to have [the monthly payments], but I will make it work.”*
Comprehensive vs. minimal changes in food spending		LI reduced the quantity and quality of food purchases to a greater extent than HI	*“I often make sure I have a set amount that I spend biweekly when I get my paychecks to make sure [food] is still something that is constantly in the house. The times when I do not get paid, I try and limit what I buy to cheaper options, meals that last longer, and eliminate snacks.”*	*“Before, I was able to buy a little more [food] to put up until later, but now, I buy what I need, and use what I need.”*
Presence vs. absence of barriers to medical care		LI experienced cost barriers to medical care that HI did not	*“It is not just getting the time off. It is making sure I actually have the funds that day to pay for the co-pay.”*	*“We have health insurance through work, so for the most part, [doctor’s visits] are affordable after it comes out of our paycheck.”*
**4. Overall views on ECTC**					
Desire for ECTC to continue	LI felt ECTC monthly payments were helpful to them. HI felt they were helpful to others	*“I wish [the monthly payments] would come back, and I wish they would come back as soon as possible. I wish they would think about what that means to parents, and how helpful it is at a time like this.”*	*“We are fortunate that we are not struggling for anything basic. If it helped us when we did not really need it for essentials, then I cannot imagine how much it is helping people who really need it. So, I am sad to see it go.”*		
Anticipated financial security vs. flexibility	LI predicted a greater ability to afford basic needs. HI predicted financial flexibility.	*“I think we would definitely be in a much better space where, even if we were still partially behind, it would not be as bad.”*	*“We would have the extra money to get the things she wants done. Not so much the things she needs, but some of the things she would like.”*		

a Families earning < 200% Federal Poverty Line;

b Families earning > 200% Federal Poverty Line

## Abbreviations

CTC: Child Tax Credit
ECTC: Expanded Child Tax Credit
HI: high income
LI: low income
